# Association study of *AGO1* and *AGO2* genes polymorphisms with recurrent pregnancy loss

**DOI:** 10.1038/s41598-019-52073-0

**Published:** 2019-10-30

**Authors:** Young Ran Kim, Chang Soo Ryu, Jung Oh Kim, Hui Jeong An, Sung Hwan Cho, Eun Hee Ahn, Ji Hyang Kim, Woo Sik Lee, Nam Keun Kim

**Affiliations:** 1 Department of Obstetrics and Gynecology, CHA Bundang Medical Center, CHA University, 65th Street, Yatap Road, Bundang-gu, Seongnam 13497 South Korea; 2 Department of Biomedical Science, College of Life Science, CHA University, 335 Pangyo-ro, Bundang-gu, Seongnam 13488 South Korea; 3 Fertility Center of CHA Gangnam Medical Center, CHA University, 566 Nonhyeon-ro, Gangnam-gu, Seoul 06135 South Korea

**Keywords:** Genetic association study, Genetic markers

## Abstract

An Argonaute (AGO) protein within the RNA-induced silencing complex binds a microRNA, permitting the target mRNA to be silenced. We hypothesized that variations in AGO genes had the possibility including affected the miRNA function and associated with recurrent pregnancy loss (RPL) susceptibility. Especially, we were chosen the *AGO1* (rs595961, rs636832) and *AGO2* (rs2292779, rs4961280) polymorphisms because of those polymorphisms have already reported in other diseases excluding the RPL. Here, we conducted a case-control study (385 RPL patients and 246 controls) to evaluate the association of four polymorphisms with RPL. We found that the *AGO1* rs595961 AA genotype, recessive model (*P* = 0.039; *P* = 0.043, respectively), the *AGO1* rs636832 GG genotype, and recessive model (*P* = 0.037; *P* = 0.016, respectively) were associated with RPL in women who had had four or more consecutive pregnancy losses. The patients with the *AGO1* rs636832 GG genotypes had greater platelet counts (*P* = 0.023), while the patients with the *AGO2* rs4961280 CA genotypes had less homocysteine (*P* = 0.027). Based on these results, we propose that genetic variations with respect to the *AGO1* and *AGO2* genotypes are associated with risk for RPL, and might serve as useful biomarkers for the prognosis of RPL.

## Introduction

Recurrent pregnancy loss (RPL) is defined by multiple, consecutive pregnancy loss. Some experts consider the consecutive loss of two pregnancies as RPL, whereas others restrict the definition of RPL to three or more losses. Depending on which definition is applied, it is estimated that 2–4% of women attempting to become pregnant experience^1^. Despite a considerable amount of recent progress identifying the causes of RPL, the underlying factors remain unknown for approximately 50% of RPL cases^2^. Among the possible causes are cytogenetic abnormalities, antiphospholipid syndrome, anatomical factors, and hormonal factors, but it appears that few cases are caused by a single pathogenic factor. Rather, most cases are attributed to an interaction of more than one genetic risk factor. The uncertainty about the causes of RPL has made it difficult to identify the genes and biological mechanisms involved. Using a candidate gene approach in tandem with genome-wide association studies, researchers have been able to identify low- and moderate-penetrance alleles associated with RPL risk. Although the identified risk variants cannot fully explain the RPL heritability, these variants of the genetic area were an important discovery^3^. Furthermore, there is accumulating evidence of differential association between specific genetic factors and distinct RPL subtypes^4–6^.

The microRNA (miRNA) is a 21- to 24-bp non-coding RNA that base pairs with its complementary sequences in an mRNA and silences the mRNA by several possible mechanisms. MiRNAs are important in many pathological conditions and aberrant miRNA expression can have serious consequences. Aberrant miRNAs have been associated with both RPL and endometriosis^7^ and recent studies revealed that miRNAs, which are associated with poor pregnancy outcomes, exist in bodily fluids of women and are secreted by culture-grown cells^8^. Furthermore, it has been reported that mature miRNA present in exosomes are associated with the development of autoimmune diseases such as Hashimoto’s disease and Graves’ disease^9^. In additions, miRNAs are well-known regulators of cell cycle progression, proliferation, and differentiation in the endometrium during the menstrual cycle^10–12^. All of this evidence supports a role for miRNAs in reproductive processes. The regulation of miRNA biogenesis and processing is mediated by various proteins, such as Drosha, Dicer, Exportin 5 (XPO5), DGCR8, and proteins of the Argonaute (AGO) family, along with several enzymes^13^. Pre-miRNAs are synthesized in the nucleus and are then transported by XPO5 and Ran-GTP into the cytoplasm where they are truncated by the RNase Dicer to form miRNA duplexes^14,15^. These duplexes then interact with AGO proteins present within the RNA-induced silencing complex (RISC), resulting in the formation of a functional RISC. Within the RISC, the two strands of the miRNA duplex are divided; one strand is degraded and the other acts as a template to direct binding and subsequent silencing of the target mRNA^16,17^.

The human AGO family is separated into four subfamilies. The only subfamily with catalytic activity is *AGO2*, and this subfamily serves a critical function within the RISC^18^. The RNA and proteins associated with *AGO1* and *AGO2* are present at considerable levels in many body tissues, which previously led us to focus on these two subfamilies^19^. *AGO1* inhibits the proliferation and motility of cell through inducing apoptosis^20^, and regulates genes that influence growth, survival, and the cell cycle progression^21^. In contrast, *AGO2* has been shown to be upregulated in numerous cancers and is associated with the growth of tumor cells and overall patient survival^22^. In a mouse model study, *Ago2* regulated protein expression in mouse embryos, and this had important effects on the progression of blastocyst differentiation^23^. Furthermore, deletions of both *Ago1* and *Ago2* affect the formation and cleavage activity of RISC, and the deletion of *Ago2* is associated with down-regulation of miRNAs in other tissues^24^. Overall, these findings reveal that miRNAs may be important for a successful pregnancy and the AGO protein is central to the functioning of miRNAs. Therefore, we hypothesized that the AGO protein is a susceptibility factor for RPL, as disruption of the AGO protein would disrupts miRNA function.

Here, we examined the associations of *AGO1* and *AGO2* gene polymorphisms with RPL pathogenesis and prognosis in a Korean population. Specifically, we examined two polymorphisms each for *AGO1* (rs595961, rs636832) and *AGO2* (rs2292779, rs4961280) because these polymorphisms have been studied previously and are already reported to be associated with other diseases. To our knowledge, this study is the first to provide evidence that *AGO1* and *AGO2* polymorphisms play a role in RPL of Korean women.

## Results

### The baseline characteristics

The baseline characteristics of the RPL patients and controls are shown in Table 1. The hematocrit, platelet count (PLT), and estradiol concentration (E2) in the RPL patients were greater than in the control group controls (*P* = 0.001, *P* = 0.003, *P* = 0.002, respectively). The concentration of follicle-stimulating hormone, concentration of luteinizing hormone, and prothrombin time were all significantly different between RPL patients and members of the control group (all *P* < 0.0001). No other significant differences were observed in the parameters mentioned.

**Table 1:** Baseline characteristics between RPL patients and controls.

Characteristic	Controls (n = 246)	RPL patients (n = 385)	*P* ^*a*^
Age (years, mean ± SD)	33.32 ± 5.74	33.21 ± 4.55	0.792
Hematocrit (μmol/L, mean ± SD)	35.68 ± 4.25	37.31 ± 3.37	0.001^b^
PLT (10^3^/µL, mean ± SD)	236.41 ± 65.58	255.43 ± 59.22	0.003
PT (sec, mean ± SD)	11.53 ± 3.10	11.58 ± 0.86	<0.0001^b^
aPTT (sec, mean ± SD)	32.51 ± 4.16	32.24 ± 4.33	0.636
BMI (kg/m^2^, mean ± SD)	21.66 ± 3.33	21.49 ± 3.84	0.673
BUN (mg/dL, mean ± SD)	8.39 ± 1.77	9.99 ± 2.77	0.061
Creatinine (mg/dL, mean ± SD)	0.69 ± 0.08	0.72 ± 0.12	0.400
Uric acid (mg/dL, mean ± SD)	4.27 ± 0.95	3.80 ± 0.84	0.340
Total cholesterol (mg/dL, mean ± SD)	311.00 ± 140.01	187.73 ± 49.42	0.066^b^
Folate (nmol/L, mean ± SD)	12.16 ± 9.43	14.21 ± 11.94	0.613
Homocysteine (μmol/L, mean ± SD)	7.79 ± 1.38	6.98 ± 2.10	0.249
FSH (mIU/mL, mean ± SD)	8.12 ± 2.85	7.52 ± 10.52	<0.0001^b^
LH (mIU/mL, mean ± SD)	3.32 ± 1.74	6.30 ± 12.09	<0.0001^b^
E2 (pg/mL, mean ± SD)	26.00 ± 14.75	35.71 ± 29.46	0.002^b^
TSH (µIU/mL, mean ± SD)	—	2.18 ± 1.55	—
Prolactin (ng/mL, mean ± SD)	—	15.68 ± 12.98	—
CD56 NK cell (%, mean ± SD)	—	18.26 ± 7.99	—
Triglyceride (mg/dL, mean ± SD)	—	181.42 ± 156.63	—
HDL-chol (mg/dL, mean ± SD)	—	61.82 ± 17.63	—
FBS (mg/dL, mean ± SD)	—	95.24 ± 16.97	—

*P*^*a*^ was calculated using a two-sided t-test for continuous variables. ^b^We were calculated using the Mann-Whitney test for continuous data when F-test *P*-value for equal variances was lower than 0.05. RPL, recurrent pregnancy loss; PLT, platelet; PT, prothrombin time; aPTT, activated partial thromboplastin time; BMI, body mass index; BUN, blood urea nitrogen; FSH, follicle-stimulating hormone; LH, luteinizing hormone; E2, estradiol; TSH, thyroid stimulating hormone; HDL-chol, high-density lipoprotein cholesterol; FBS, fasting blood sugar; SD, standard deviation.

### Genotype frequency analyses of the *AGO1* and *AGO2* gene polymorphisms between RPL patients, subgroups of RPL patients, and controls

To confirm that depending on the increasing number of pregnancy losses was associated with *AGO1* and *AGO2* gene polymorphisms, the patient subgroup was further divided into two groups based on a number of pregnancy losses. The first group is that had three or more pregnancy loss (PL) (subgroup PL ≥ 3), and the second group is that had four or more PL (subgroup PL ≥ 4). We investigated the *AGO1* polymorphisms rs595961G>A and rs636832A>G, and the *AGO2* polymorphisms rs2292779C>G and rs4961280C>A, in all groups. The results are shown in Table 2. The genotype frequencies of the polymorphisms were satisfied in Hardy-Weinberg equilibrium (*P* > 0.05). The *AGO1* polymorphisms rs595961G>A and rs636832A>G was associated with prevalence of RPL prevalence in the subgroup PL ≥ 4 (Table 2). These two polymorphisms were significantly associated with RPL under the recessive model (*AGO*1 rs595961 GG + GA vs. AA: AOR = 4.008, 95% CI = 1.047–15.349, *P* = 0.043; rs636832 AA + AG vs. GG: AOR = 2.821, 95% CI = 1.210–6.577, *P* = 0.016). However, we did not detect a significant association between RPL and the polymorphisms in total RPL patients (PL ≥ 2) or and the subgroup (PL ≥ 3).

**Table 2:** Genotype frequency analyses of polymorphisms of genes for the Argonaute proteins *AGO1* and *AGO2* in RPL patients, subgroups of patients with RPL and controls.

Genotype	Controls (n = 246)	RPL patients (n = 385)	AOR (95% CI)	*P* ^*^	PL ≥ 3 (n = 203)	AOR (95% CI)	*P* ^*^	PL ≥ 4 (n = 81)	AOR (95% CI)	*P* ^*^
*AGO1* rs595961G>A										
GG	189 (76.8)	275 (71.4)	1.000 (reference)		146 (71.9)	1.000 (reference)		57 (70.4)	1.000 (reference)	
GA	53 (21.5)	96 (24.9)	1.231 (0.838–1.807)	0.289	49 (24.1)	1.204 (0.772–1.879)	0.413	19 (23.5)	1.187 (0.650–2.169)	0.577
AA	4 (1.6)	14 (3.6)	2.412 (0.782–7.442)	0.126	8 (3.9)	2.576 (0.761–8.724)	0.128	5 (6.2)	4.146 (1.075–15.996)	0.039
Dominant (GG vs GA + AA)			1.313 (0.907–1.901)	0.150		1.300 (0.849–1.991)	0.228		1.396 (0.796–2.448)	0.244
Recessive (GG + GA vs AA)	2.295 (0.746–7.054)	0.147		2.464 (0.731–8.308)	0.146		4.008 (1.047–15.349)	0.043		
HWE-*P*	0.898	0.130								
*AGO1* rs636832A>G										
AA	126 (51.2)	218 (56.6)	1.000 (reference)		113 (55.7)	1.000 (reference)		42 (51.9)	1.000 (reference)	
AG	107 (43.5)	138 (35.8)	0.729 (0.521–1.018)	0.064	73 (36.0)	0.765 (0.517–1.131)	0.180	28 (34.6)	0.783 (0.455–1.349)	0.378
GG	13 (5.3)	29 (7.5)	1.277 (0.641–2.547)	0.487	17 (8.4)	1.456 (0.677–3.130)	0.337	11 (13.6)	2.547 (1.061–6.118)	0.037
Dominant (AA vs AG + GG)	0.788 (0.572–1.086)	0.146		0.840 (0.578–1.221)	0.361		0.975 (0.589–1.611)	0.920		
Recessive (AA + AG vs GG)	1.455 (0.741–2.858)	0.276		1.627 (0.770–3.437)	0.202		2.821 (1.210–6.577)	0.016		
HWE-*P*	0.108	0.276								
*AGO2* rs2292779C>G										
CC	92 (37.4)	156 (40.5)	1.000 (reference)		86 (42.4)	1.000 (reference)		38 (46.9)	1.000 (reference)	
CG	125 (50.8)	174 (45.2)	0.825 (0.584–1.165)	0.275	89 (43.8)	0.763 (0.511–1.140)	0.187	35 (43.2)	0.678 (0.398–1.154)	0.152
GG	29 (11.8)	55 (14.3)	1.135 (0.675–1.910)	0.633	28 (13.8)	1.065 (0.584–1.943)	0.837	8 (9.9)	0.674 (0.282–1.610)	0.375
Dominant (CC vs CG + GG)	0.880 (0.633–1.224)	0.449		0.817 (0.558–1.194)	0.296		0.676 (0.407–1.122)	0.130		
Recessive (CC + CG vs GG)	1.246 (0.770–2.018)	0.371		1.217 (0.696–2.125)	0.491		0.819 (0.358–1.873)	0.636		
HWE-*P*	0.170	0.565								
*AGO2* rs4961280C>A										
CC	216 (87.8)	321 (83.4)	1.000 (reference)		171 (84.2)	1.000 (reference)		66 (81.5)	1.000 (reference)	
CA	30 (12.2)	59 (15.3)	1.325 (0.827–2.126)	0.242	29 (14.3)	1.238 (0.715–2.146)	0.446	14 (17.3)	1.526 (0.763–3.055)	0.232
AA	0 (0.0)	5 (1.3)	NA	0.998	3 (1.5)	NA	0.998	1 (1.2)	NA	0.998
Dominant (CC vs CA + AA)	1.438 (0.902–2.294)	0.127		1.366 (0.797–2.340)	0.256		1.639 (0.830–3.235)	0.155		
Recessive (CC + CA vs AA)	NA	0.998		NA	0.998		NA	0.998		
HWE-*P*	0.230	0.233								

RPL, recurrent pregnancy loss; PL, pregnancy loss; AOR, adjusted odds ratio; AGO, Argonaute; HWE, Hardy-Weinberg equilibrium; 95% CI, 95% confidence interval; ^*^Adjusted by age.

### Allele combinations analysis of *AGO1* and *AGO2* polymorphisms in RPL patients and control women

Table 3 shows allele combination models and the frequencies in which they were observed in the RPL and control groups. We analyzed allele combinations for all four polymorphism and observed an association between seven allele combinations (G-A-C-A, G-A-G-C, G-G-G-C, A-A-C-C, A-A-G-C, A-G-C-C, A-G-G-C) and RPL risk (Table 3). Among them, the combinations G-A-C-A (AOR = 3.705), G-A-G-C (AOR = 1.347), A-G-C-C (AOR = 4.137), and A-G-G-C (AOR = 5.736) had an increased association with RPL prevalence compared to the control group, while the other allele combination models had a decreased association with RPL compared to the control group. Furthermore, this tendency held for allele combination analysis of two and three polymorphisms. Particularly, when the allele combination included the minor allele of *AGO1* rs595961G>A and rs636832A>G, we observed increased association with RPL (Table 3). For example, all of the combinations A-G-C (*AGO1* rs595961/*AGO1* rs636832/*AGO2* rs2292779; AOR = 3.903, 95% CI = 2.122–7.179, *P < *0.0001), A-G-C (*AGO1* rs595961/*AGO1* rs636832/*AGO2* rs4961280; AOR = 3.796, 95% CI = 2.260–6.375, *P* < 0.0001), and A-G (*AGO1* rs595961/*AGO1* rs636832; AOR = 2.961, 95% CI = 1.839–4.767, *P* < 0.0001) had increased odds ratios for RPL risk compared to the control subjects. In addition, we performed a combination analysis that compared genotype combination frequencies in RPL patients and control subjects (Supplementary Table S2).

**Table 3:** Analyses of allele combinations of *AGO1* and *AGO2* polymorphisms in RPL patients and controls.

Allele combinations	Controls (2n = 492)	RPL patients (2n = 770)	OR (95%CI)	*P*
*AGO1* rs595961G>A/*AGO1* rs636832A>G/*AGO2* rs2292779C>G/*AGO2* rs4961280C>A				
G-A-C-C	223 (45.4)	311 (40.3)	1.000 (reference)	
G-A-C-A	6 (1.3)	31 (4.0)	3.705 (1.520–9.032)	0.002
G-A-G-C	107 (21.8)	201 (26.1)	1.347 (1.007–1.802)	0.045
G-A-G-A	14 (2.9)	22 (2.9)	1.127 (0.564–2.251)	0.735
G-G-C-C	37 (7.6)	52 (6.8)	1.008 (0.639–1.589)	0.974
G-G-C-A	2 (0.5)	4 (0.5)	1.434 (0.260–7.901)	1.000
G-G-G-C	40 (8.1)	22 (2.8)	0.394 (0.228–0.682)	0.001
G-G-G-A	0 (0.0)	3 (0.4)	5.022 (0.258–97.790)	0.270
A-A-C-C	20 (4.1)	5 (0.6)	0.179 (0.066–0.485)	0.0003
A-A-C-A	4 (0.9)	1 (0.1)	0.179 (0.020–1.616)	0.167
A-A-G-C	16 (3.2)	4 (0.5)	0.179 (0.059–0.544)	0.001
A-A-G-A	1 (0.3)	0 (0.0)	0.239 (0.010–5.903)	0.419
A-G-C-C	13 (2.7)	75 (9.8)	4.137 (2.240–7.641)	<0.0001
A-G-C-A	1 (0.3)	8 (1.0)	5.736 (0.712–46.210)	0.089
A-G-G-C	4 (0.9)	32 (4.2)	5.736 (2.000–16.460)	0.0002
*AGO1* rs595961G>A/*AGO1* rs636832A>G/*AGO2* rs2292779C>G				
G-A-C	206 (41.9)	341 (44.3)	1.000 (reference)	
G-A-G	114 (23.2)	224 (29.1)	1.187 (0.893–1.577)	0.237
G-G-C	64 (13.1)	56 (7.3)	0.529 (0.355–0.787)	0.002
G-G-G	47 (9.5)	25 (3.2)	0.321 (0.192–0.538)	<0.0001
A-A-C	25 (5.1)	5 (0.7)	0.121 (0.046–0.321)	<0.0001
A-A-G	14 (2.8)	3 (0.5)	0.130 (0.037–0.456)	0.001
A-G-C	13 (2.7)	84 (10.9)	3.903 (2.122–7.179)	<0.0001
A-G-G	9 (1.8)	31 (4.1)	2.081 (0.971–4.459)	0.055
*AGO1* rs595961G>A/*AGO1* rs636832A>G/*AGO2* rs4961280C>A				
G-A-C	330 (67.1)	512 (66.5)	1.000 (reference)	
G-A-A	21 (4.4)	53 (6.9)	1.627 (0.963–2.747)	0.067
G-G-C	79 (16.0)	74 (9.6)	0.604 (0.427–0.853)	0.004
G-G-A	1 (0.2)	7 (0.9)	4.512 (0.552–36.860)	0.160
A-A-C	35 (7.2)	8 (1.1)	0.147 (0.067–0.322)	<0.0001
A-A-A	6 (1.2)	0 (0.1)	0.050 (0.003–0.884)	0.004
A-G-C	18 (3.6)	106 (13.8)	3.796 (2.260–6.375)	<0.0001
A-G-A	2 (0.3)	9 (1.2)	2.900 (0.623–13.510)	0.218
*AGO1* rs595961G>A/*AGO2* rs2292779C>G/*AGO2* rs4961280C>A				
G-C-C	260 (52.8)	364 (47.2)	1.000 (reference)	
G-C-A	9 (1.8)	35 (4.5)	2.778 (1.312–5.879)	0.006
G-G-C	149 (30.2)	222 (28.8)	1.064 (0.819–1.382)	0.641
G-G-A	14 (2.9)	26 (3.3)	1.327 (0.679–2.590)	0.406
A-C-C	33 (6.7)	79 (10.3)	1.710 (1.105–2.646)	0.015
A-C-A	7 (1.5)	9 (1.1)	0.918 (0.338–2.498)	0.868
A-G-C	20 (4.2)	36 (4.7)	1.286 (0.728–2.272)	0.386
*AGO1* rs595961G>A/*AGO1* rs636832A>G				
G-A	320 (65.1)	565 (73.4)	1.000 (reference)	
G-G	111 (22.5)	81 (10.5)	0.413 (0.301–0.568)	<0.0001
A-A	39 (7.9)	9 (1.2)	0.131 (0.062–0.273)	<0.0001
A-G	22 (4.5)	115 (15.0)	2.961 (1.839–4.767)	<0.0001
*AGO1* rs595961G>A/*AGO2* rs2292779C>G				
G-C	270 (55.0)	398 (51.7)	1.000 (reference)	
G-G	161 (32.7)	248 (32.3)	1.045 (0.813–1.344)	0.732
A-C	39 (7.9)	88 (11.5)	1.531 (1.018–2.301)	0.040
A-G	22 (4.5)	36 (4.6)	1.110 (0.639–1.929)	0.711
*AGO1* rs595961G>A/*AGO2* rs4961280C>A				
G-C	408 (83.0)	586 (76.1)	1.000 (reference)	
G-A	23 (4.6)	60 (7.8)	1.816 (1.105–2.986)	0.017
A-C	54 (10.9)	115 (14.9)	1.483 (1.048–2.098)	0.026
A-A	7 (1.5)	9 (1.2)	0.895 (0.331–2.424)	0.827

RPL, recurrent pregnancy loss; AGO, Argonaute; 95% CI, 95% confidence interval; OR, odds ratio.

### Differences in clinical traits with respect to *AGO1* and *AGO2* polymorphisms

**Figure 1:**
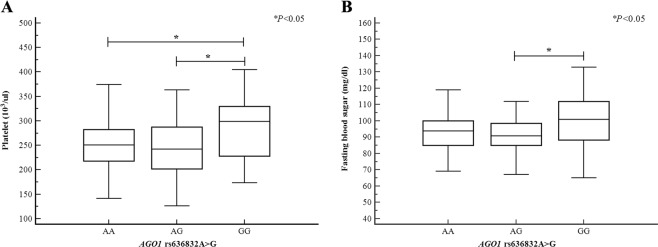
Association between platelet counts, fasting blood sugar levels, and *AGO1* rs636832A>G polymorphisms in patients with RPL. (**A**) Patients with the *AGO1* rs636832 GG genotype had significantly higher platelet levels than patients with the *AGO1* rs636832 AA genotype or AG genotype. (**B**) Patients with the *AGO1* rs636832 AG genotype had significantly flower fasting blood sugar levels than patients with the *AGO1* rs636832 GG genotype. Abbreviation: RPL, recurrent pregnancy loss.

We measured the platelet count of peripheral blood, fasting blood sugar, the hematocrit, body mass index, plasma levels of fasting total homocysteine, and triglyceride levels (Table 4). The platelet counts and fasting blood sugar level were elevated in RPL patients with the *AGO1* rs636832 GG genotype compared to the *AGO1* rs636832 AA and AG genotypes (Fig. 1). The hematocrit was lower in control individuals with the *AGO1* rs636832 GG and *AGO2* rs2292779 GG genotypes compared to the other genotypes. Also, the plasma triglyceride levels were elevated in patients with the *AGO2* rs4961280 AA genotype (Table 4). Other factors were not associated with the polymorphisms examined here.

**Table 4:** Clinical parameters of individuals in the study with different genotypes with respect to *AGO1* and *AGO2* polymorphisms.

Control					Patients						
Genotypes	Hct (%)	PLT(10^3^/ul)	BMI (kg/m2)	Hcy (μmol/L)	Genotypes	Hct (%)	PLT (10^3^/ul)	BMI (kg/m2)	Hcy (μmol/L)	TG (mg/dl)	FBS (mg/dl)
	Mean ± SD	Mean ± SD	Mean ± SD	Mean ± SD		Mean ± SD	Mean ± SD	Mean ± SD	Mean ± SD	Mean ± SD	Mean ± SD
*AGO1* rs595961G>A					*AGO1* rs595961G>A						
GG	35.62 ± 4.21	233.03 ± 64.52	21.47 ± 2.96	8.38 ± 1.31	GG	37.40 ± 3.12	252.52 ± 51.74	21.33 ± 3.30	6.94 ± 2.00	191.00 ± 167.11	94.30 ± 14.12
GA	35.71 ± 4.57	250.77 ± 69.72	22.14 ± 4.44	7.18 ± 1.55	GA	37.41 ± 3.67	266.31 ± 76.47	22.25 ± 5.23	7.11 ± 2.36	168.20 ± 145.56	94.63 ± 16.80
AA	38.83 ± 1.72	210.67 ± 65.42	—	—	AA	35.09 ± 5.04	237.63 ± 55.74	19.60 ± 2.00	6.94 ± 2.13	132.50 ± 55.60	119.00 ± 43.32
*P*^*a*^	0.436	0.238	0.379	0.281	*P* ^*a*^	0.164	0.491^b^	0.028	0.842	0.706	0.089^b^
*AGO1* rs636832A>G					*AGO1* rs636832A>G						
AA	35.09 ± 4.42	233.36 ± 69.62	21.81 ± 3.47	8.10 ± 1.79	AA	37.28 ± 3.13	252.97 ± 53.04	21.21 ± 2.93	6.96 ± 1.97	196.44 ± 179.46	94.91 ± 15.03
AG	36.79 ± 3.71	241.24 ± 61.27	21.38 ± 3.12	7.23 ± 0.68	AG	37.71 ± 3.52	250.89 ± 61.04	22.08 ± 5.15	6.83 ± 2.14	147.07 ± 103.47	93.15 ± 14.72
GG	34.63 ± 5.20	243.38 ± 52.04	19.35 ± 1.25	—	GG	35.69 ± 4.00	294.06 ± 80.67	20.75 ± 2.53	7.96 ± 2.61	224.33 ± 187.00	107.06 ± 31.04
*P*^*a*^	0.026^b^	0.704	0.417	0.655^b^	*P* ^*a*^	0.093	0.023	0.084	0.079	0.670^b^	0.044^b^
*AGO2* rs2292779C>G					*AGO2* rs2292779C>G						
CC	35.68 ± 4.64	232.55 ± 62.67	21.48 ± 3.03	7.36 ± 1.27	CC	37.19 ± 3.74	254.61 ± 64.40	21.13 ± 2.97	7.00 ± 2.04	209.00 ± 174.22	96.07 ± 18.74
CG	36.24 ± 3.79	244.22 ± 64.62	21.86 ± 3.92	8.47 ± 1.79	CG	37.27 ± 3.20	248.93 ± 50.39	21.94 ± 4.39	7.04 ± 2.28	152.59 ± 135.21	93.96 ± 16.32
GG	33.29 ± 4.49	215.53 ± 77.13	21.39 ± 2.55	—	GG	37.75 ± 2.73	275.34 ± 64.30	20.97 ± 4.15	6.75 ± 1.60	203.33 ± 172.12	96.70 ± 13.51
*P*^*a*^	0.011	0.138	0.819	0.342	*P* ^*a*^	0.726	0.096	0.117	0.769	0.339	0.629
*AGO2* rs4961280C>A					*AGO2* rs4961280C>A						
CC	35.75 ± 4.39	235.79 ± 66.96	21.57 ± 3.09	7.98 ± 1.44	CC	37.19 ± 3.38	255.96 ± 59.07	21.53 ± 3.86	7.13 ± 2.14	169.60 ± 149.42	95.76 ± 17.67
CA	35.23 ± 3.28	243.27 ± 57.69	22.01 ± 4.82	7.43 ± 1.79	CA	37.70 ± 3.27	250.82 ± 61.82	20.97 ± 3.68	6.17 ± 1.70	207.55 ± 151.97	93.11 ± 13.76
AA	—	—	—	—	AA	40.95 ± 1.48	289.50 ± 2.12	24.26 ± 5.01	6.18 ± 0.74	603.00 ± 0.00	90.00 ± 7.07
*P*^*a*^	0.591	0.618	0.636	0.649	*P* ^*a*^	0.222	0.646	0.162	0.027	0.017	0.635

ANOVA, analysis of variance; SD, standard deviation; Hct, Hematocrit; PLT, platelet; Hcy, Homocysteine; BMI, body mass index; TG, Triglyceride; FBS, fasting blood sugar. ^a^Calculated using ANOVA. ^b^We were calculated using the Kruskal-Wallis test for continuous data when F-test *P*-value for equal variances was lower than 0.05.

## Discussion

RPL is defined repeated consecutive spontaneous abortions. Important factors involved in recurrent early pregnancy loss are genetic, endocrine, anatomical, and immunological in origin^25^. At present, the correlation between RPL and genetic polymorphisms that have been identified with next-generation sequencing is unclear, although genetic polymorphisms have been noted to confer susceptibility to RPL^26^. In this study, the association between four polymorphisms of *AGO1* and *AGO2* genes and the susceptibility for RPL in a Korean population was examined, specifically *AGO1* rs595961G>A, *AGO1* rs636832A>G, *AGO2* rs2292779C>G, and *AGO2* rs4961280C>A. These four polymorphisms were significantly associated with the RPL patient group and the association between the polymorphisms and RPL can be explained by mechanisms other than protein expression: the polymorphism may affect the splicing of precursor mRNA and the conformation and function of proteins associated with this process^27^; or the polymorphisms may affect the structure of AGO proteins rather than their quantity. We believe the present study is the first to reveal a relationship between susceptibility to RPL and *AGO1* and *AGO2* polymorphisms.

The miRNAs have been implicated in numerous pathological conditions^13^ and in the regulation of biochemical pathways, including cell proliferation, differentiation, metabolism, apoptosis, development, inflammation, and immunity in many eukaryotic organisms^28,29^. The mature miRNA in exosomes has been associated with immune regulation^30^. Previous studies identified an association between *AGO1* and *AGO2* and an angiogenesis defect model associated with the inflammation^31^. Furthermore, AGO1 was associated with an angiogenic pathway involving hypoxia-responsive miRNAs that could be a potentially suitable target for anti- or pro-angiogenesis^32^. Importantly, the regulation of AGO2 is reportedly the safety mechanism that limits the range of the anti-inflammatory activity of miR-146a^33^. Our previous study also demonstrated an association between miR-146a and risk of recurrent implantation failure^34^. As noted, AGO2 is the catalytic core of mammalian RISC involved in miRNA expression, and is reportedly essential to mammalian gastrulation and mesoderm formation^35–38^. Since, it is well-known that different miRNAs can bind to distinct sequences caused by polymorphisms^39^. Interestingly, the expression of vascular endothelial growth factor (VEGF) was significantly correlated with the expression of AGO2 protein^40^. Therefore, there is a well-established link between *AGO1* and *AGO2* and RPL, based in part on the relationship between AGO proteins, immune regulation, and autoimmune diseases^30,41^.

In accord with our hypothesis, our analyses revealed that the *AGO1* rs595961G>A and *AGO1* rs636832A>G genotypes were significantly associated with the prevalence of RPL in this study. Several factors associated with RPL, specifically, a patient’s hematocrit, platelet count, body mass index, homocysteine, triglyceride levels, and fasting blood sugar have been shown to be significantly associated with *AGO1* and *AGO2* gene polymorphisms in this study. Interestingly, in a recent survey, it was concluded that that there are no differences in effective factors between two and three or more consecutive pregnancy losses^42^. However, we found associations between *AGO1* and *AGO2* polymorphisms and number of RPLs. Specifically, the occurrence of four or more PLs was associated with *AGO1* rs595961G>A and *AGO1* rs636832A>G polymorphisms.

In the present study, we found that the *AGO1* rs595961G>A genotype was associated with decreased body mass index in RPL patients. Moreover, the *AGO2* rs4961280C>A genotype was associated with the decreased hematocrit in RPL patients. The previous studies were demonstrated that Ago2 had mediated the functions in the control of hematopoiesis in the mouse model and hematopoietic stem cell model, and was reported that had functioned as a critical regulator of erythropoiesis in the mouse model^43,44^. These result and previous studies were showed the possibility that this polymorphism of *AGO2* might affect the process of hematopoiesis or erythropoiesis. Also, the *AGO1*rs636832A>G and *AGO2*rs2292779C>G genotypes were associated with the lower homocysteine levels in RPL patients. Furthermore, patients with the *AGO1* rs636832GG genotype had significantly higher platelet counts than patients with the *AGO1* rs636832AA or AG genotypes (Fig. 1A). In addition, patients with the *AGO1*rs636832AG genotype had significantly lower fasting blood sugar levels than patients with the *AGO1*rs636832GG genotype (Fig. 1B). We conclude that the *AGO1* polymorphisms studied here are associated with RPL.

There had a few limitations, which should be considered when interpreting the results, in this study. If we were progressed the functional study of other polymorphisms of Argonaute in the future, we will gain more powerful evidence. However, we have focused on the study that would be finding the biomarkers of RPL, and we have progressed that associated the study between various polymorphism of Argonaute located in intron variants and the prevalence of RPL. Therefore, the functional study of these polymorphisms of Argonaute is not possible to the progressing because of these polymorphisms were located in the intron variants. Moreover, the study groups were small because we relied on a single medical center and selected participants based on strict criteria. Despite these limitations, we detected statistically significant differences between the patient and control groups. If our results are confirmed in larger, multicenter studies, the regulation of *AGO1* and *AGO2* may serve as a biomarker to the diagnosis of RPL.

In conclusion, we have identified associations between polymorphisms in the *AGO1* gene and prevalence of RPL in a Korean population, as well as the significant clinical effects. These polymorphisms of *AGO1* gene were located to intron variant but showed that have the possibility of biomarkers to diagnose and estimate the risk of RPL. Furthermore, we suggest that these polymorphisms may be associated with the pathogenesis of RPL.

## Methods

### Study population

The population in this study comprised 631 participants. The control and patient groups met strict criteria. The 246 women in the control group were recruited at the CHA Bundang Medical Center. Each control subject had a normal (46, XX) karyotype, regularly occurring menstrual cycles, a history of one or more pregnancies that were conceived naturally, and no prior history of pregnancy losses. The 385 women in the patient group were diagnosed with RPL between March 1999 and December 2012 at the Department of Obstetrics and Gynecology of CHA Bundang Medical Center, CHA University (Seongnam, Republic of Korea)^45^. RPL was defined as a minimum of two consecutive losses of pregnancy, and pregnancy loss was diagnosed by various clinical tests, namely, ultrasound, human chorionic gonadotropin testing, or physical examination prior to 20 weeks gestation^2^. RPL subgroups were assigned according to the number of pregnancy losses. The study excluded patients with pregnancy losses caused by infectious, hormonal, anatomical, autoimmune, chromosomal, or thrombotic factors. Anatomical abnormalities were determined using sonography, hysterosalpingogram, hysteroscopy, magnetic resonance imaging, or computed tomography. Hormonal causes of RPL, such as thyroid disease, luteal insufficiency, and hyperprolactinemia, were identified by measuring peripheral blood levels of follicle-stimulating hormone, luteinizing hormone, prolactin, thyroid-stimulating hormone, progesterone, and free T4. The autoimmune factors lupus and antiphospholipid syndrome were assessed using lupus anticoagulant and anti-cardiolipin antibodies, respectively. Thrombophilia, a thrombotic factor for RPL, was evaluated from levels of anti-β2 glycoprotein antibody and deficiencies in proteins C and S. A total of the 481 patients were initially evaluated for the study, but 96 patients exhibited trisomy, hypothyroidism, intrauterine adhesion, antiphospholipid syndrome, or chromosomal translocation (patients or spouses), and were therefore excluded from the study, leaving 385 subjects in the patient group^5^. This study was approved by the CHA Bundang Medical Center Institutional Review Board (IRB) and the Hospital Ethics Committee (IRB No. BD2010-123D). All study protocols abided by the recommendations of the Declaration of Helsinki and written informed consent was obtained from all study participants.

### Evaluation of clinical factors in control and patient samples

Blood samples of RPL patients were obtained for a period of pregnancy and were used to measure blood coagulation factors, urate concentration, total cholesterol, and levels of folate, and homocysteine after fasting for 12 hours. Homocysteine levels were measured on an Abbott IMx analyzer (Abbott Laboratories, Abbott Park, IL, USA) using a fluorescence polarization immunoassay^46^. Folate, creatinine, and blood urea nitrogen (BUN) levels were measured by competitive immunoassay using ACS:180 (Bayer Diagnostics, Tarrytown, NY, USA)^46^. Urate and total cholesterol levels were measured with enzymatic colorimetric tests (Roche Diagnostics, Mannheim, Germany). An automated photo-optical coagulometer (ACL TOP; Mitsubishi Chemical Medicine, Tokyo, Japan) was used to measure prothrombin time (PT), and activated partial thromboplastin time (aPTT), and a Sysmex XE2100 automated hematology analyzer (Sysmex, Kobe, Japan) was used to determine platelet counts^46^. Hormone levels were measured in one of two ways, depending on the hormone; radioimmunoassays were used to measure estradiol, thyroid-stimulating hormone and prolactin (Beckman Coulter, Inc., Brea, CA, USA), and enzyme immunoassays were used to measure follicle-stimulating hormone and luteinizing hormone (Siemens AG, Munich, Germany)^47^. The proportion of the CD56+ natural killer cells proportion in peripheral blood was determined by flow cytometry (FACS Calibur; BD Biosciences, USA)^48^.

### Genotyping

A Genomic DNA Extraction Kit (iNtRON Biotechnology, Seongnam, Republic of Korea) was used to extract genomic DNA from the peripheral blood collected from patients and controls. We examined the following four polymorphisms in this study: *AGO1* rs595961G>A, *AGO1* rs636832A>G, *AGO2* rs2292779C>G, and *AGO2* rs4961280C>A. The classification of alleles of the genetic polymorphism was confirmed to the East Asian population on the 1000Genomes study. The polymorphisms were assessed using polymerase chain reaction-restriction fragment length polymorphism (PCR-RFLP) with the following primers: *AGO1* rs595961G>A, forward 5′-CCC TAC ATC CAG GAA TTT GGG-3′ and reverse 5′-TCG ACA CTG TTT TTG GGG TG-3′; *AGO1* rs636832G>A, forward 5′-CTG ATT CCA GAA CAT ATC ACT CAT-3′ and reverse 5′-GGT ATA CCC AGA GAC TGA AAG TAA A-3′; *AGO2* rs2292779C>G, forward 5′-CGG AAC AAG CAG TTC CAC AC-3′ and reverse 5′-TGA CAG GGA AAG GCT GAT GA-3′; and *AGO2* rs4961280C>A, forward 5′-TGC CCC TGT CTC CTT CAC ATG TCC-3′ and reverse 5′-GTT CCC CAA CAC AGC GCT CAA AGG-3′. The four polymorphisms were confirmed by digestion with restriction enzymes *Nla*III *Aci*I, *Bfa*I, and *Hpy*166II (New England Bio Laboratories, MA, USA) for 16 hours at 37 °C. For the *AGO1* rs595961G>A polymorphism, the wild-type genotype GG was confirmed by the presence of one 349 bp band after enzymatic digestion, the heterozygous genotype GA was confirmed by the detection of three bands at 349, 203, and 146 bp after enzymatic digestion, and the mutant genotype AA was confirmed by the detection of two bands at 203 and 146 bp after enzymatic digestion. For the *AGO2* rs2292779C>G polymorphism, the wild-type genotype CC was confirmed by the presence of two bands at 142 and 99 bp, the heterozygous genotype CG was confirmed by the presence of three bands at 241, 142, and 99 bp, and the mutant genotype GG was confirmed by the presence of a single band at 241 bp. For the *AGO1* rs636832G>A polymorphism, the presence of two bands at 96 bp and 25 bp were indicative of the wild-type genotype GG, three bands at 121 bp, 96 bp, and 25 bp were indicative of the heterozygous genotype GA, and a single band at 121 bp were indicative of the mutant genotype AA. The digestion fragments for the *AGO2* rs4961280C>A polymorphism differed only slightly between genotypes; the wild-type genotype CC produced only one band at 169 bp, and the heterozygous genotype CA produced three band at 169 bp, 146 bp, and 23 bp, and the mutant genotype AA produced two band at 146 bp and 23 bp. Information regarding the PCR primers, restriction enzymes, and genotype-specific fragment sizes for each polymorphism is provided in Supplementary Table S1. Genotypes were determined by resolving the digestion fragments on 4% agarose gels using electrophoretic separation. A random 30% of the PCR product for each AGO polymorphism was used in a duplicate PCR assay, the product of which was subjected to DNA sequencing on an ABI 3730xl DNA Analyzer (Applied Biosystems, Foster City, CA, USA) to verify the RFLP results. There was 100% concurrence between the RFLP and sequencing results for each sample.

### Statistical analysis

The frequencies of the *AGO1* and *AGO2* polymorphisms were compared between the patient and control groups using logistic regression analysis and Fisher’s exact test. Adjusted odds ratio (AOR) and 95% confidence intervals (CIs) were determined from age-adjusted logistic regression and were used to evaluate the association between each polymorphism and RPL incidence. Additionally, the 95% CI and AOR were used to evaluate the association of each polymorphism and allele combination. A *P*-value < 0.05 was deemed to indicate statistical significance. None of the polymorphisms examined in this study deviated from Hardy-Weinberg equilibrium (*P* > 0.05). Statistical analyses were conducted with following software: GraphPad Prism 4.0 (GraphPad Software, Inc., San Diego, CA, USA); HAPSTAT 3.0 (University of N. Carolina, Chapel Hill, NC, USA)^5^; and StatsDirect statistics software version 2.4.4 (StatsDirect Ltd., Altrincham, UK).

## Supplementary information

Supplementary Table S1, Supplementary Table S2

## Data Availability

The data that support the findings of this study are available from the corresponding author, N. K. K., upon reasonable request.
