# Supplementary material for: Association study of AGO1 and AGO2 genes polymorphisms with recurrent pregnancy loss
Source: Sci Rep. 2019 Oct 30;9:15591. doi: 10.1038/s41598-019-52073-0 (PMC6821863; doi:10.1038/s41598-019-52073-0)
Supplement: Supplementary file 1 — Supplementary Table S1, Supplementary Table S2 [file 41598_2019_52073_MOESM1_ESM.pdf]

Supplementary Information

Title: Association study of *AGO1* and *AGO2* genes polymorphisms with recurrent pregnancy loss

Young Ran Kim [2,†], Chang Soo Ryu [1,†], Jung Oh Kim[1], Hui Jeong An[1], Sung Hwan Cho[1], Eun Hee Ahn[2], Ji Hyang Kim[2], Woo Sik Lee[3] and Nam Keun Kim[1,*]

[1]Department of Biomedical Science, College of Life Science, CHA University, 335 Pangyo-ro, Bundang-gu, Seongnam 13488, South Korea

[2]Department of Obstetrics and Gynecology, CHA Bundang Medical Center, CHA University, Seongnam 13496, South Korea

[3]Fertility Center of CHA Gangnam Medical Center, CHA University, Gangnam, 06135, South Korea

[†] Young Ran Kim and Chang Soo Ryu contributed equally to this work

[*] **Correspondence to**:

Nam Keun Kim, PhD

Department of Biomedical Science, College of Life Science, CHA University. 335 Pangyo-ro, Bundang-gu, Seongnam 13488, South Korea

Tel: +82-31-881-7137

Fax: +82-31-881-7249

E-mail: nkkim@cha.ac.kr

Running title: *AGO1* and *AGO2* polymorphisms and RPL risk

Keywords: Argonaute, microRNA, recurrent pregnancy loss, polymorphism

Supplementary Table S1. Information of *AGO1, AGO2* polymorphisms for PCR-RFLP.

Gene	Rs number	CHR	Position	Primer sequence	Probe sequence	Annealing temperature	Restriction enzyme	Genotypes size
AGO1	rs595961	ch1	35902179	F: 5'- CCC TAC ATC CAG GAA TTT GGG -3'<br>R: 5'- TCG ACA CTG TTT TTG GGG TG -3'		58℃	*Bfa*I	GG: 349bp<br>GA: 349bp, 203bp, 146bp<br>AA: 203bp, 146bp
AGO1	rs636832	ch1	35897874	F: 5'- CTG ATT CCA GAA CAT ATC ACT CAT -3'<br>R: 5'- GGT ATA CCC AGA GAC TGA AAG TAA A -3'		55℃	*Nla*III	GG: 96bp, 25bp<br>GA: 121bp, 96bp, 25bp<br>AA: 121bp
AGO2	rs2292779	ch8	140551294	F: 5'- CGG AAC AAG CAG TTC CAC AC -3'<br>R: 5'- TGA CAG GGA AAG GCT GAT GA -3'		58℃	*Aci*I	CC: 142bp, 23bp<br>CG: 165bp, 142bp, 23bp<br>GG: 165bp
AGO2	rs4961280	ch8	140637315	F: 5’ - TGC CCC TGT CTC CTT CAC ATG TCC – 3’<br>R: 5’ - GTT CCC CAA CAC AGC GCT CAA AGG – 3’		58℃	*Hpy*166II	CC: 169bp<br>CA: 169bp, 146bp, 23bp<br>AA: 146bp, 23bp

PCR-RFLP, polymorphism chain reaction-restriction fragment length polymorphism; AGO, argonaute; CHR, chromosome.

Supplementary Table S2. Combined genotypes analysis of *AGO1* and *AGO2* polymorphisms in RPL patients and controls

Combined genotypes	Controls (n=246)	RPL patients (n=385)	AOR (95%CI)	*P*[*]
AGO1 rs595961G>A/*AGO1* rs636832A>G				
GG/AA	126 (51.2)	213 (55.3)	1.000 (reference)	
GG/AG	57 (23.2)	54 (14.0)	0.560 (0.364 - 0.864)	0.009
GG/GG	6 (2.4)	8 (2.1)	0.787 (0.267 - 2.321)	0.664
GA/AA	29 (11.8)	5 (1.3)	0.101 (0.038 - 0.267)	<0.0001
GA/AG	20 (8.1)	81 (21.0)	2.363 (1.380 - 4.044)	0.002
GA/GG	4 (1.6)	10 (2.6)	1.498 (0.460 - 4.881)	0.503
AA/AA	2 (0.8)	0 (0.0)	NA	0.998
AA/AG	2 (0.8)	3 (0.8)	0.868 (0.143 - 5.273)	0.878
AA/GG	0 (0.0)	11 (2.9)	NA	0.998
AGO1 rs636832A>G/*AGO2* rs4961280C>A				
AA/CC	135 (54.9)	182 (47.3)	1.000 (reference)	
AA/CA	22 (8.9)	34 (8.8)	1.145 (0.641 - 2.047)	0.648
AA/AA	0 (0.0)	2 (0.5)	NA	0.998
AG/CC	72 (29.3)	112 (29.1)	1.144 (0.790 - 1.658)	0.477
AG/CA	7 (2.8)	23 (6.0)	2.419 (1.008 - 5.804)	0.048
AG/AA	0 (0.0)	3 (0.8)	NA	0.997
GG/CC	9 (3.7)	27 (7.0)	2.239 (1.019 - 4.918)	0.045
GG/CA	1 (0.4)	2 (0.5)	1.539 (0.138 - 17.192)	0.726
GG/AA	0 (0.0)	0 (0.0)	NA	NA

RPL, recurrent pregnancy loss; AGO, Argonaute; 95% CI, 95% confidence interval; AOR, adjusted odds ratio; NA, not applicable. [*] Adjusted by age
